# Semaphorin-7A promotes macrophage-mediated mammary epithelial and ductal carcinoma in situ invasion

**DOI:** 10.21203/rs.3.rs-6448305/v1

**Published:** 2025-05-15

**Authors:** Petra A Dahms, Brendan Hinckley, Rytis Prekeris, Fariba Behbod, Traci R Lyons

**Affiliations:** University of Colorado Anschutz Medical Campus; University of Colorado Anschutz Medical Campus; University of Colorado Anschutz Medical Campus; University of Kansas Medical Center; University of Colorado Anschutz Medical Campus

**Keywords:** SEMA7A, DCIS, macrophage, matrix-remodeling, invasion, ECM

## Abstract

**Background:**

Ductal carcinoma in situ (DCIS) accounts for 20–30% of all breast cancer diagnoses. Considered stage 0, DCIS is contained in the ducts by the myoepithelium that surround the luminal cells in the mammary gland. DCIS can progress to invasive ductal carcinoma (IDC) if the tumor cells break through the myoepithelium and invade the surrounding breast tissue. While 30–50% of DCIS tumors will progress to IDC, a majority will remain in a DCIS-like state. The mechanisms that drive this progression are not completely understood. There is currently no clinically recognized biomarker for predicting risk of DCIS progression. Therefore, all DCIS tumors are treated with standard of care, resulting in overtreatment. We have previously identified independent roles for semaphorin-7A (SEMA7A) and collagen in promoting DCIS progression to IDC.

**Methods:**

To investigate the relationship between SEMA7A and collagen remodeling in the mammary gland, we utilized patient tissues and mouse models of normal development and DCIS progression as well as a novel SEMA7A-blocking antibody.

**Results:**

We show that SEMA7A increases in patient samples of DCIS compared to matched normal tissues and in IDC compared to matched DCIS and normal tissues. This increase was correlated with the presence of CD68 + macrophages. Using puberty in the mammary gland as a model for normal epithelial invasion facilitated by macrophages, we show SEMA7A knockout mice exhibit delayed ductal elongation as well as decreased macrophages. Additionally, our SEMA7A-blocking antibody in a mouse model of DCIS decreased invasive tumor phenotypes and decreased organized collagen around the tumor. The invasive tumors had increased collagen and macrophage influx in the tumor. Finally, we show that SEMA7A activates an AKT/GSK3β/β-catenin signaling pathway within macrophages to promote expression of pro-inflammatory cytokines and the matrix remodeling enzyme MMP9 to facilitate invasion.

**Conclusions:**

Our results demonstrate that SEMA7A regulates normal and transformed epithelial cell invasion through regulation of pro-invasive matrix remodeling via macrophages. Our studies also suggest that SEMA7A expression, macrophage phenotype, and collagen structure may be a predictor of risk for DCIS invasion. Thus, blocking SEMA7A may be a novel therapeutic strategy for high-risk DCIS patients to slow or prevent progression of disease.

## Background

Breast cancer is the leading cancer diagnosis in women in the United States (US), making up an estimated 15.5% of all new cancer cases in 2024 (1). Additionally, the rate of new breast cancer cases is steadily increasing (2). Thus, it is crucial to evaluate treatment and care for breast cancer patients. Approximately 20–25% of newly diagnosed breast cancers are ductal carcinoma in situ (DCIS)(3), known as “stage 0” or pre-invasive breast cancer (4, 5), with an estimated 56,500 cases diagnosed in 2024 (1). Routine screening has resulted in increased detection and diagnosis of DCIS in the US (6, 7). While many tumors will remain in a DCIS-like state, DCIS can progress to invasive ductal carcinoma (IDC). IDC is the most common type of breast cancer that is characterized by cancer cells invading outside the ducts and into the surrounding breast tissue (8). However, only 30–50% of DCIS cases will progress to IDC over a patient’s lifetime (7, 9–11); yet, DCIS patients are provided the same standards of care as other breast cancers, including surgery and radiation. suggesting that many patients are overtreated (12, 13). Survival outcomes are better for patients diagnosed with regions of both IDC and DCIS when compared to IDC alone (14). DCIS also has an approximate 20% recurrence rate, with half of recurrences diagnosed as invasive (15). Therefore, providing care that could reduce DCIS progression may also improve prognosis by reducing the risk of invasive recurrence.

DCIS develops through hyperproliferation of epithelial cells that line the mammary ducts and is characterized by encapsulation by the myoepithelium (16). The myoepithelium is a specialized cell layer that surrounds the normal mammary ducts and separates the luminal epithelial cells from the surrounding stroma (17, 18). It is suggested that gaps in, or loss of, the myoepithelium leads to cancer cell invasion into the surrounding stroma resulting in IDC. What causes the myoepithelium to break is not completely understood and there is no clinical biomarker for predicting progression. Published findings suggest that the tissue/tumor microenvironment (TME) of the mammary gland—specifically immune and stromal cells found in the mammary gland—could contribute to progression of DCIS to IDC (19–23). As such, it is important to understand the relationship between DCIS and the TME.

We have previously shown that DCIS progression to IDC can be influenced by normal mammary remodeling during postpartum mammary gland involution. Postpartum involution is the process where the milk secreting epithelial cells undergo cell death after pregnancy and/or lactation, which is accompanied by matrix remodeling, allowing for the mammary gland to return to a pre-pregnant state poised for subsequent lactation(s). The process of postpartum involution results in a “wound-healing” environment where increased collagen deposition promotes increased cyclooxygenase-2 (COX-2) expression and DCIS invasion (22). We identified a signaling molecule, semaphorin-7A (SEMA7A), that is both increased during mammary gland involution and regulated by COX-2 (24, 25). SEMA7A is a glycosylphosphatidylinositol (GPI)-anchored signaling molecule that can be shed from the cell surface to promote autocrine and paracrine signaling (26–28). Our previous results indicate that SEMA7A is both necessary and sufficient to promote DCIS invasion (24, 27). However, how SEMA7A signaling from DCIS contributes to regulation of pro-invasive stromal phenotypes has not been explored.

Macrophages are important regulators of both normal mammary development and the TME, making up 40% of immune cells in breast tumors where they can mediate cell invasion, immune suppression, and vasculogenesis (29, 30). Macrophages are found increased in DCIS tissues when compared to normal tissues and remained high in IDC tissues (31). Macrophages in the stroma also predicted for shorter time to recurrence and invasive recurrence (32, 33). One well characterized function of macrophages during mammary gland morphogenesis, specifically during puberty and postpartum involution, is matrix remodeling (30, 34–39), and matrix remodeling is known to facilitate tumor cell invasion (40–43). These parallels between invasion and remodeling in the mammary fat pad during mammary morphogenesis and IDC have supported that studies of pubertal development of the mammary gland could provide insight into general mechanisms of epithelial cell invasion that could be hijacked by tumor cells, specifically during DCIS progression (44–46). These include macrophage-mediated matrix remodeling, collagen organization that provides directional cues for forward ductal invasion, and the coordinated regulation of cell proliferation and invasion (16, 21, 30, 37, 47–53). During puberty, macrophages accumulate around the main proliferative and invasive structures, or terminal end buds (TEBs), and along the elongated ducts (37) to aid in collagen structure organization and are necessary for proper ductal elongation (38). Once puberty is complete, the epithelial-associated macrophages become resident mammary macrophages (37). Additionally, collagen alignment is associated with a pathologically poorer prognosis (54) and is increased in recurrent DCIS (55).

For our studies presented herein, we utilized pubertal development as a model of normal epithelial cell invasion in the mammary gland to gain insight into the role(s) of SEMA7A in DCIS invasion. To test our hypothesis that SEMA7A mediates matrix remodeling in the mammary gland and during DCIS progression via regulation of macrophages, we utilized SEMA7A knockout mice to analyze pubertal development and a function blocking antibody against SEMA7A in a mouse model of DCIS progression. Our results identify a SEMA7A-mediated signaling mechanism that promotes protease production and activity in macrophages to facilitate collagen remodeling and DCIS invasion.

## Methods

### Cell Culture

MCF10DCIS.com (and SEMA7A overexpression and GFP derivatives) were cultured in 2D and 3D cultures as previously described (27). RAW264.7 macrophages were obtained from the laboratory of R. Nemonoff (CU Anschutz Medical Campus, Aurora, CO) and cultures as previously described (35)(O’Brien 2010). THP-1 cells were gifted from J. Richer (CU Anschutz Medical Campus, Aurora, CO). THP-1 cells were cultured in suspension in T75 flasks in RPMI media with 10% FBS and 1% penicillin-streptomycin. THP-1 cells were treated with 1ug/mL phorbol 12-myristate-13-acetate (PMA) for 24 hours for differentiation prior to treatments. Cells were validated and identified as a pure population of respective cell lines by the DNA sequencing core at the CU Anschutz Medical Campus. Cells were regularly tested for mycoplasma throughout studies. Overexpression plasmid (p304-V5-Blasticidin and V5-SEMa7A) were obtained from the Functional Genomics Core at the CU Anschutz Medical Campus and overexpression was confirmed via qPCR and Western blot analysis. GFP derivatives were created by transduction with a viral vector (pLVX-AcGFP) purchased from Invitrogen and expression was confirmed via immunofluorescence. Purified SEMA7A was isolated in collaboration with the Protein Purification/MoAB/Tissue culture core at the CU Anschutz Medical Campus from MDA-MB-231 cells engineered to overexpress SEMA7A via an overexpression plasmid (SEMA7A-Fc) as a gift from from R. Medzhitov (Yale University, New Haven, CT) with the control plasmid (pcDNA3.1) was obtained from H. Ford (CU Anschutz Medical Campus, Denver CO).

### Data Mining

*SEMA7A* and *CD68* gene expression analysis in insitu carcinomas vs invasive carcinomas from the Early Stage Breast Tumors (SCAN-B) dataset was performed on http://ualcan.path.uab.edu/index.html sorting by biopsy type (56).

### Tissue Microarray & Analysis

Tissue microarrays containing normal, DCIS, and IDC samples were prepared from biopsy tissue following placement in preservation media and storage at 4C, as previously described (24, 57). Tissues were stained for SEMA7A and CD68. Antibody information is in Supplement Table 1. Slides were scanned into Aperio Digital Pathology Imaging Systems using Aperio ScanScope3 (Leica Biosystems, Deer Park, IL, USA). SEMA7A and CD68 were analyzed with an optimized color analysis algorithm using MATLAB software as described below. Change in SEMA7A expression level determined by ± 1% difference between Normal, DCIS, and IDC % area value. Change in CD68 expression level determined by any up or down difference between Normal, DCIS, and IDC % area value. All analyzable tissue biopsies were included as points for expression analysis, but only paired tissues were included in the statistical analysis and denoted as lines connecting pairs in the graph. For DCIS tissues, Normal, n = 34; DCIS, n = 29; paired, n = 28. For DCIS + IDC tissues, Normal, n = 86; DCIS, n = 80; IDC, n = 85. Paired **MATLAB Analysis**

SVS image files from Aperio were loaded into the MATLAB 2024b (MathWorks, Natick, MA, USA) workspace. A custom threshold for SEMA7A and CD68 was established and saved for processing. The images underwent a series of morphological operations and texture filtration steps to isolate the biopsies from the surrounding slide background. To reduce computational load, the tissue was segmented into blocked regions, which were processed sequentially. Each block was analyzed for SEMA7A and CD68, with small noise artifacts removed before storing the extracted values in the corresponding location within the final output. The function generated a labelmap for validation and visualization, along with pixel quantity measurements for SEMA7A and CD68 in each biopsy. Normal to DCIS, n = 72; paired DCIS to IDC, n = 74; paired Normal to IDC, n = 77.

### Rodent breeding for puberty study

*Sema7a* ^tm1Alk^/J mice (Jackson Laboratories) and C57Bl/6 mice were housed and bred as previously described (25, 58). Briefly, *Sema7a*^tm1Alk^/J mice were a generous gift from Alex Kolodkin at John’s Hopkins University. The age-matched wild-type control C57Bl/6 mice were bred at the CU Anschutz Medical Campus or purchased from Jackson Labs. Mice were sacrificed for the study based on weekly (7 day) increments from date of birth, with a one-day window before or after. #4 right and left mammary glands were harvested from female Sema7atm1Alk/J and age-matched wild type C57Bl/6 female mice. Mammary glands were prepared for whole mount or formalin-fixed, paraffin-embedded for immunohistochemistry analysis.

### Whole mount staining of mouse mammary gland

The fourth mammary gland was harvested from mice and spread out on a regular microscope slide. The gland was immediately fixed in Carnoy’s fix (60% ethanol, 30% chloroform, 10% glacial acetic acid) for 24 hours at room temperature followed by an optional 70% ethanol short-term storage. Gland was then rehydrated in EtOH baths at 50, 30, and 10% EtOH twice in order for 10 minutes each; washed in distilled H2O for 5 minutes; and rinsed with PBS. Gland was stained in carmine alum solution (1g carmine, 2.5g aluminum potassium sulfate, in 500mL diH2O; boiled for 20 minutes and filtered) overnight at room temperature. The following day, gland was dehydrated in 70, 95, and 100% EtOH baths twice in order for 15 minutes each. Glands were then cleared in 100% xylene for at least one hour, or until noticeable change. Cleared gland were imaged by light microscopy and stored long-term in methyl salicylate.

### Whole mount analysis

Ductal elongation past LN was measured by imaging glands alongside a ruler in millimeters. Branch end counts and widths were quantified via ImageJ (National Institutes of Health and Laboratory for Optical and Computational Instrumentation, University of Wisconsin, Bethesda, MD, USA). TEBs were determined by a width cut-off of 2.5 pixels as being a larger than average standard width.

### Immunohistochemistry and immunofluorescence

Mammary glands with and without tumor were prepared as previously described (22). Antibody information is provided in Supplement Table 1. 5μm tissue sections were stained and imaged at 10X magnification using Olympus microscope (Olympus Scientific Solutions, Evident Scientific, Walthan, MA, USA). Analysis of staining was performed using CellSens Imaging and Analysis software (Olympus Scientific Solutions). Briefly, areas of stroma, tumor border, and tumor were annotated using CellSens and quantification of Ki67, trichrome, F4/80, and αSMA. Percent positive was calculated as area with positive stain (positive pixels) divided by total area (total pixels) and multiplied by 100. For immunofluorescence imaging of β-catenin, 500,000 RAW264.7 macrophages were seeded on collagen-coated glass cover slips and treated with 40μg/mL SEMA7A for 48 hours. Cells were then fixed in 4% PFA and probed with active β-catenin antibody. 5 representative area per treatment were imaged using 63X magnification on Zeiss microscope (Carl Zeiss Microscopy, White Plains, NY, USA) as z-stack images. Images were processed and analyzed with ImageJ. Each z-stack was processed as a max projection with background subtracted with 10 pixel radius to eliminate background signal. 30 nuclei per image were analyzed for percent positive area, object count (foci), mean intensity, and integrated density, and averaged for each image.

### Animal tumor model

The MCF10DCIS tumor model was utilized as previously described (27). Briefly, 6–8 week old female SCID Hairless Outbred (SHO) mice from Charles River were injected with 250,000 MCF10DCIS-GFP cells in 50uL PBS into the right and left #4 mammary fat pads. Tumors were measured 2–3 times weekly via GFP signal using Illumatool Tunable Lighting System technology (Lightools Research). Mice were administered 250μg/50μL SmAbH1 or control immunoglobulin antibody IgG1 by IP injection every third day. Weights were taken every treatment day and mice were monitored for weight loss and health/behavior changes by University OLAR and lab staff. Mice were sacrificed at 4 weeks post-initial injection. All animal studies were approved by the IACUC of University of Colorado, Anschutz Medical Campus.

### Histological tumor analysis

Tissue for tumor invasion analysis were prepared for hematoxylin and eosin and blinded for analysis as previously described (27). Briefly, evaluations of *in situ* were determined based on morphologic phenotype. Well-contained lesions with a clear basement membrane were scored as 0; less defined borders with minimum basement membrane and micro-invasions were scored as 1; increased invasive components including no basement membrane, immune cell infiltration, and increased vasculature in the lesion with a defined border were scored as 2; lesions that were mixed with stromal components with no defined border were scored as 3.

### Second harmonic generation microscopy

5μm unstained tissues were imaged at the UC Anschutz Advanced Light Microscopy Core Facility using a Zeiss LSM 780 Confocal Microscope (Carl Zeiss Microscopy, White Plains, NY, USA). 3 representative mammary glands with tumors were imaged from each treatment and matched by invasion score. 2–5 representative images were taken of the tumor border and surrounding stroma for analysis. SHG images were analyzed for intensity over distance using ImageJ with averaged 3 measurements of 100um away from the tumor border. SHG images were analyzed for orientation and alignment using CurveAlign for Fibrillar Collagen Quantification (Laboratory for Optical and Computational Instrumentation, University of Wisconsin-Madison, Madison, WI, USA)(59). For CurveAlign, boxes were drawn around the stroma region next to the tumor and analyzed with Curvelets method and 0.06 fractions of coefs.

### In situ zymography (Dye-quenched (DQ) collagen assay)

In situ zymography (DQ gelatin) assays were adapted from a previously described protocol (60, 61). Mammary gland tissues containing tumor were fixed with 70% EtOH for 48 hours before processing tissue sections as previously described (22). After sectioning, tissues were processed with xylene twice for 10 minutes and rehydrated in 100, 95, and 70% EtOH twice in order for 5 minutes each. Tissues were then incubated with DQ gelatin from Pig Skin, Fluorescein Conjugate (Invitrogen, Cat #D12054). DQ gelatin was dissolved at 1mg/mL. Tissues were incubated with 1:50 DQ-gelatin for 4 hours before washing twice with PBS for 5 minutes and mounted with media containing DAPI (Vectashield, #H-1200-10). Tissues were then imaged at 10X magnification on Olympus microscope (Olympus Scientific Solutions) using FITC and DAPI filter. For analysis using CellSens software, a100um × 200um ROI was fit over a region on the tumor border and analyzed for percent positive area and mean intensity; object counting was performed manually.

### 3D Organoid Invasion Assay

3D organoid invasion assay was adapted previously described protocol (Lyons 2011). Briefly, 5,000 MCF10DCIS.com were cultured in 100uL 4mg/mL Matrigel (Corning, Kennebunk, ME, USA) with or without 10% 4mg/mL rat tail collagen type I (Corning), and containing 0 or 40ug/mL SEMA7A and/or 500 RAW264.7 macrophages. Conditions were made in triplicate. Images were taken on Olympus microscope (Olympus Scientific Solutions, Evident Scientific, Walthan, MA, USA) at 4 days post-seeding. Z-stack images were taken of each replicate and each organoid was analyzed for invasion as invasive or non-invasive. Invasive organoids were then taken as a percentage of total organoids in the z-stack images.

### Cytokine Array

Cytokine secretion in conditioned media was measured via the commercially available kit for human cytokines (Bio-techne, R&D Systems, ARY022B) according to manufacturer instructions. Arrays were imaged on a LI-COR Odyssey CLx imager (LI-COR Biosciences, Lincoln, NE, USA).

### Western Blot

Western blots were performed as previously described (24). Protein was harvested from fresh cell lysates and concentration was determined by Bradford or Qubit Protein Broad Range Assay. Equal amounts of protein were loaded into each lane. Antibody information is available in Supplement Table 1. Blots were imaged and quantitated on a LICOR Odyssey ClX Imager (LiCOR).

### qPCR

RNA was isolated, processed as cDNA, and qPCR of cDNA was performed as previously described (24). RNA and cDNA concentration was determined by Nanodrop. QPCR was performed using Bio-Rad iTaq Universal SYBR Green Supermix (Bio-Rad #1725121). GAPDH was used as reference gene. All primers except Human COX2 were obtained from IDT Technologies (Coralville, IA, USA) with the following sequences: COX2 (Mouse, F:GCATTCTTTGCCCAGCACTTCACT, R:TTTAAGTCCACTCCATGGCCCAGT; TNFα (Mouse, F:GGTGCCTATGTCTCAGCCTCTT, R:GCCATAGAACTGATGAGAGGGAG; Human, F:CTCTTCTGCCTGCTGCACTTTG, R:ATGGGCTACAGGCTTGTCACTC), c-MYC (Mouse, F:TCGCTGCTGTCCTCCGAGTCC, R: GGTTTGCCTCTTCTCCACAGAC; Human, F:CCTGGTGCTCCATGAGGAGAC, R:CAGACTCTGACCTTTTGCCAGG), IL1b (Mouse, F:TGGACCTTCCAGGATGAGGACA, R:GTTCATCTCGGAGCCTGTAGTG; Human, F:CCACAGACCTTCCAGGAGAATG, R: GTGCAGTTCAGTGATCGTACAGG); IL4 (Mouse, F:ATCATCGGCATTTTGAACGAGGTC, R:ACCTTGGAAGCCCTACAGACGA; Human, F:CCGTAACAGACATCTTTGCTGCC, R:GAGTGTCCTTCTCATGGTGGCT), IL6 (Mouse, F:TACCACTTCACAAGTCGGAGGC, R:CTGCAAGTGCATCATCGTTGTTC; Human, F:AGACAGCCACTCACCTCTTCAG, R:TTCTGCCAGTGCCTCTTTGCTG), GAPDH: (Mouse, F:AAGGTCATCCCAGAGCTGAA, R:CTGCTTCACCACCTTCTTGA; Human, F:CACCTCATTCTCCTGGCTATC, R:CTGGCATCTGACTGTGTAGAA). Human COX2 primers were ordered from Bio-Rad (Unique Assay ID: qHsaCED0042341).

### Zymography Gel Assay

Zymography gel assay was performed as previously described (35). Briefly, a 7.5% SDS-PAGE gel containing 3mg/mL porcine gelatin was made fresh with equal amounts of 48-hour conditioned media loaded into each lane. After substrate buffer incubation, proteinase activity was visualized by Coomassie Blue R250 stain with activity appearing as white bands on a dark background. Images were taken by light microscopy and quantitated in ImageJ.

### Statistical Analysis

Unpaired, paired, two-tailed, and one-tailed t-tests and two-way ANOVA were performed with GraphPad Prism 10. Error bars indicate mean ± standard error of mean. A p-values < 0.05 were considered significant. All in vitro studies were performed in technical triplicate and/or biological triplicates with pooled data is shown. All in vivo studies had number of mice/group/time-point based on power calculations from previous and pilot studies to achieve at least 80% power (β) with α = 0.05. Animal tumor studies were replicated twice with representative or pooled data shown. Outliers were removed if significant by the ROUT (Q = 1%) test.

## Results

### SEMA7A increases with DCIS progression in patient samples and positively correlates with macrophage presence

**Figure 1:**
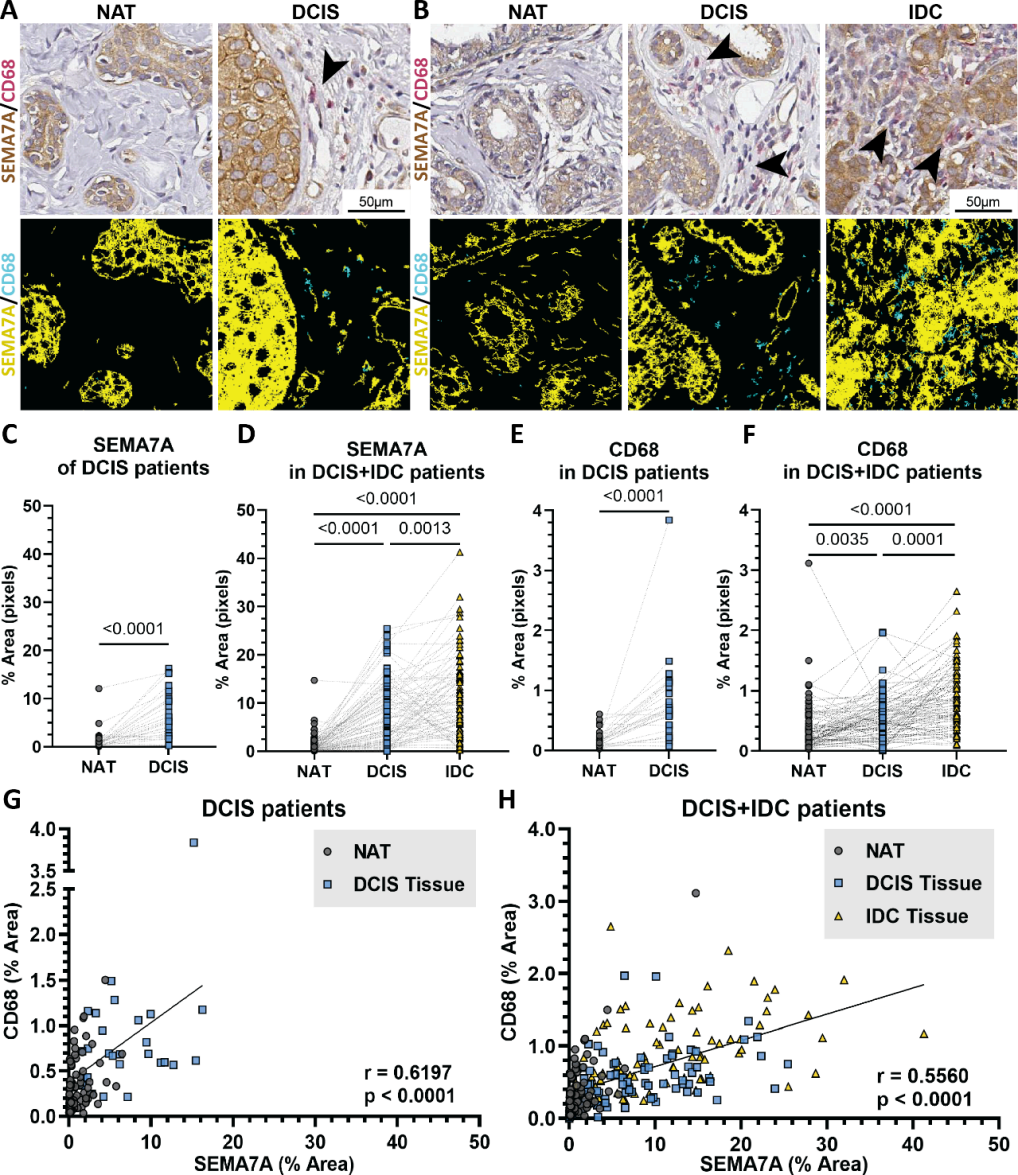
SEMA7A is positively correlated with CD68 in DCIS and IDC tissues. (A-B) (top) Representative images of NAT, DCIS, and IDC from DCIS and DCIS+IDC tissues showing SEMA7A expression (brown), CD68 expression (pink) and CD68+ cells (black arrows). (bottom) Corresponding images showing mask for SEMA7A expression (yellow), CD68 expression (cyan), and negative expression (black). (C-D) Percent positive pixels for SEMA7A and (E-F) CD68 by quantitative IHC in tumor microarrays for NAT, DCIS and IDC tissues, in patients with DCIS (TMA1) (C, D) or DCIS+IDC (TMA2)(E, F). (G-H) Correlation analysis of SEMA7A and CD68 expression evaluated in all tissues from the DCIS (TMA1) and DCIS+IDC (TMA2) patient tumor microarrays. P values determined by paired two-tailed t-test (C-F). p<000.1 by two-tailed Pearson correlation analysis (G-H).

To test our hypothesis that SEMA7A plays a role in progression of DCIS in patients, we examined SEMA7A expression in Early Stage Breast Tumors (SCAN-B) for breast cancers by biopsy type and found that SEMA7A mRNA is upregulated in invasive carcinomas compared to in situ carcinomas (Supp Fig. 1A). To expand our analysis to SEMA7A protein in patient tissues, we examined SEMA7A expression in tissue microarrays (TMA) from patient biopsies that contain DCIS only (TMA1) or IDC with histologically distinct DCIS remnants (TMA2) and compared to matched NAT (Fig. 1A–B). These two patient groups, DCIS only (TMA1) and DCIS + IDC (TMA2), were analyzed independently based on their differential diagnoses. We observed that SEMA7A increased in 85.7% of DCIS compared to NAT in TMA1 (Fig. 1A, Supp Fig. 1C); in TMA2, we observed increased SEMA7A in 86.1% of DCIS compared to NAT and increased SEMA7A in 66.7% of IDC compared to DCIS (Fig. 1D, Supp Fig. 1C). In TMA2, we also observed that 50.7% exhibited SEMA7A expression in their tissues that progressively increased from NAT to DCIS to IDC where SEMA7A had the highest expression (Supp Fig. 1C). In TMA2, 1.5% had no change in SEMA7A expression as between DCIS and IDC. The remaining 47.8% had other varying changes in SEMA7A expression across DCIS + IDC progression; for example, some tissues had increased expression between NAT and DCIS with no change between DCIS and IDC, or a decrease in SEMA7A expression from between NAT and DCIS with a larger increase between DCIS and IDC. Overall, 0% of patient tissues had a decrease in SEMA7A expression in IDC when compared to DCIS.

We also observed that CD68, a human macrophage marker, increased in invasive carcinomas compared to in situ carcinomas in the SCAN-B dataset (Supp Fig. 1B). To test the hypothesis that SEMA7A expression is associated with macrophage presence in DCIS, we analyzed CD68 positive cells in TMA1 and TMA2. We found a significant increase in 89.3% of DCIS lesions compared to NAT in TMA1 (Fig. 1A, 1E, Supp Fig. 1C) and CD68 positive cells increased in 75% of DCIS compared to NAT and 79.8% of IDC compared to DCIS in TMA2 (Fig. 1B, 1F, Supp Table 1). Moreover, in TMA2, 53.03% of DCIS + IDC tissues showed an increase in CD68 positive cells between NAT, DCIS and IDC (Supp Fig. 1C). 6.06% had decreased CD68 expression in IDC compared to DCIS. 40.91% had other varying changes to CD68 expression. We observed a positive correlation between SEMA7A and CD68 positivity in both TMAs suggesting that SEMA7A may cooperate with macrophages to mediate invasion (Fig. 1G–H).

### Loss of SEMA7A results in delayed ductal elongation during pubertal development

**Figure 2:**
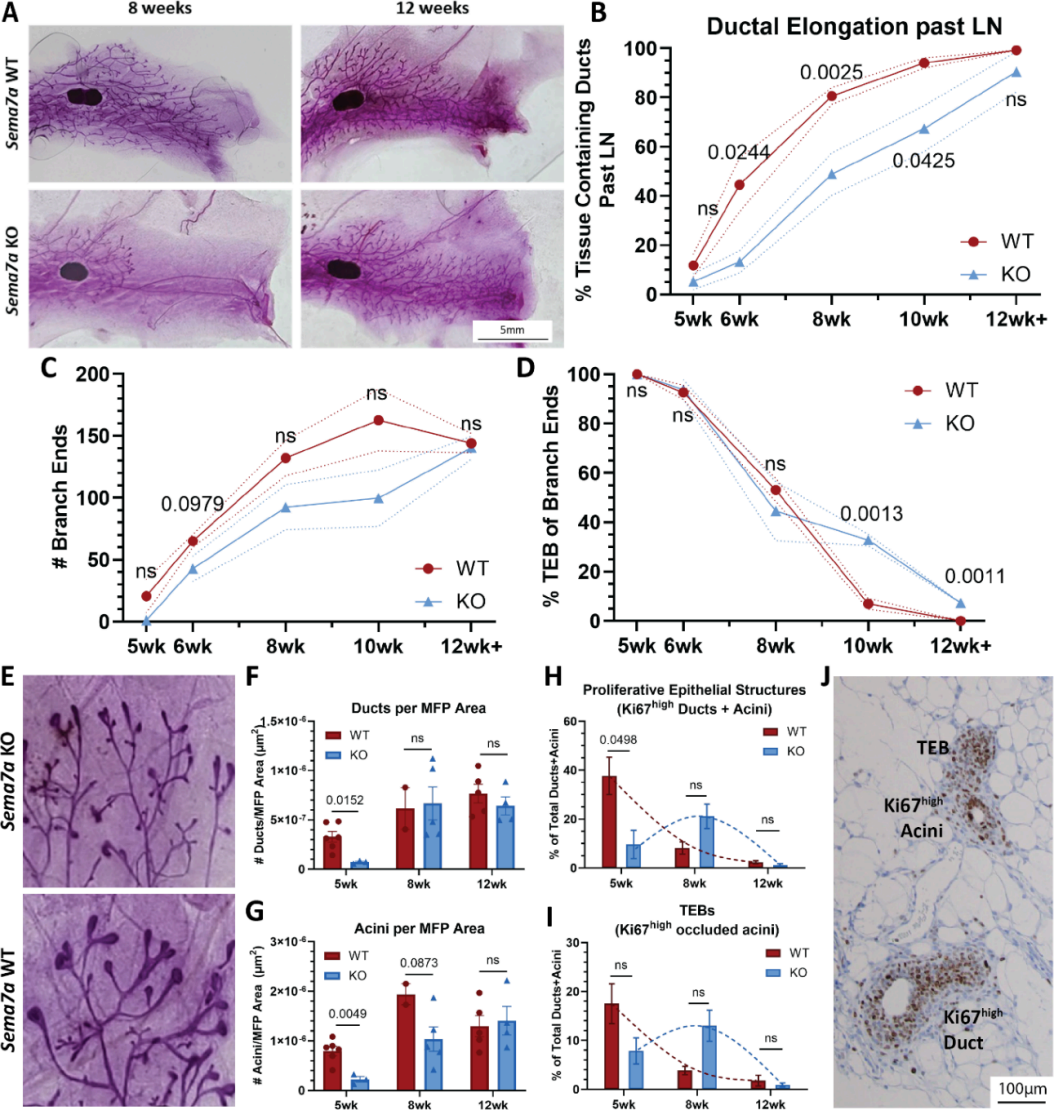
Loss of SEMA7A delays ductal elongation during pubertal development in the mammary gland. Representative whole mount images of WT (top) and KO (bottom) mice at 8 weeks (left) and 12 weeks (right). (B) Quantification of ductal elongation as a percent of tissue invasion past the LN during pubertal development timepoints. (C) Quantification of branch end points past the LN via mammary gland whole mount analysis. (D) Quantification of TEBs past the LN via mammary gland whole mount analysis. (E) Representative close-up whole mount images of TEBs at 5 weeks of age. (F) Quantification of ducts per MFP as the number of ducts by MFP area. Two-way ANOVA, p_timepoint_=0.0006; p_strain_=0.2793, p_interaction_=0.04963. (G) Quantification of acini per MFP as the number of acini by MFP area. Two-way ANOVA, p_timepoint_=0.0006; p_strain_=0.0225, p_interaction_=0.0915. (H) Quantification of TEBs in the MFP as a percent of total epithelial structures (ducts+acini). TEBs defined as occluded acini with increased proliferation via increased Ki67 expression. Two-way ANOVA, p_timepoint_=0.0042; p_strain_=0.8658, p_interaction_=0.0371. (I) Quantification of proliferative epithelial structures in the MFP via Ki67 expression as a percent of total epithelial structures (ducts+acini). Two-way ANOVA, p_timepoint_=0.0034; p_strain_=0.2843, p_interaction_=0.0106. (I) Representative image of proliferative TEB, duct, and acini in 5 week WT mammary gland. (B-C) WT, n=4–9; KO, n=5–6. (E-H) WT: 5wk, n=6; 8wk, n=2; 12wk, n=5. KO: 5wk, n=3; 8wk, n=5; 12wk, n=4. p values determined by paired two-tailed t-test (B-C, E-H).

To test our hypothesis that SEMA7A cell invasion in the normal mammary gland, we characterized pubertal development in *Sema7a* knock out (KO) mice (*Sema7a*^*tm1Alk*^*/J*). We measured ductal elongation past the lymph node in mammary gland whole mounts compared to wild-type (WT) throughout the pubertal window (Fig. 2A), starting at 5 weeks when puberty first begins (62), and at 6, 8, and 10 weeks when puberty is considered complete. We observed normal ductal elongation from 5 weeks to 10 weeks in WT mice (Fig. 2B) and a significant reduction in ductal elongation starting at 6 weeks of age in the KO mice (Fig. 2B; Supp Fig. 2A). The greatest difference was observed at 8 weeks of age (Fig. 2A–B). Because this difference remained significant at 10 weeks, we collected 12–13-week time points where we observed that both the WT and KO mice had fully formed ductal trees (Fig. 2A–B). In the whole mounts, we observed enlargement of TEBs and abnormal branching (Fig. 2E). To further investigate changes in ductal tree structure and invasion in the KO that could explain differences in phenotype, we counted total branch ends and TEBs (Supp Fig. 2B). We observed a decrease in the number of branches in the KO compared to the WT from 6–10 weeks, which was resolved at 12 weeks of age (Fig. 2C). This decrease from 6–10 weeks in the KO was also observed with the TEBs (Fig. 2D). We next examined overall size of mammary glands between WT and KO in H&E stained sections (Supp Fig. 2C) by counting the number of ducts and acini in the mammary glands. We observed decreased ducts and acini at 5 weeks in the KO mammary glands compared to WT (Fig. 2F–G), which was resolved in the KO ducts by 8 (Fig. 2F) and 12 weeks of age (Fig. 2G). Of note, there were no observable differences in overall size of the ducts and acini between the WT and KO (Supp Fig. 2D-E). To test the hypothesis that the TEB enlargement and abnormal branching was due to alterations in proliferation in the KO, we counted TEBs and proliferative epithelial structures. We defined TEB in histological sections as fully-closed, or occluded, acini with increased proliferation using nuclear Ki67 expression (Ki67^high^) as a marker (Fig. 2J). Our results identify that the number of TEBs peaks in the WT at 5 weeks, which is followed by a decrease over the remaining 2–3 weeks of the pubertal window (Fig. 2H). However, in the KO, the number of TEBs in the KOdid not peak until 8 weeks and did not decrease until 12–13 weeks (Fig. 2H). We then defined proliferative epithelial structures in histological sections as any duct or acini with increased proliferation via Ki67^high^ expression (Fig. 2J, Supp Fig. 2F). We observed highest epithelial proliferation at 5 weeks in the WT and at 8 weeks in the KO (Fig. 2I). We did not observe a significant difference between strains (WT and KO) in any proliferative structures by two-way ANOVA analysis, (Fig. 2H, p_strain_=0.8658; Fig. 2I, p_strain_=0.2843). However, two-way ANOVA analysis found a significant difference between timepoints (Fig. 2H, p_timepoint_=0.0042; Fig. 2I, p_timepoint_=0.0034).

### Delayed macrophage localization contributes to impeded ductal elongation

**Figure 3:**
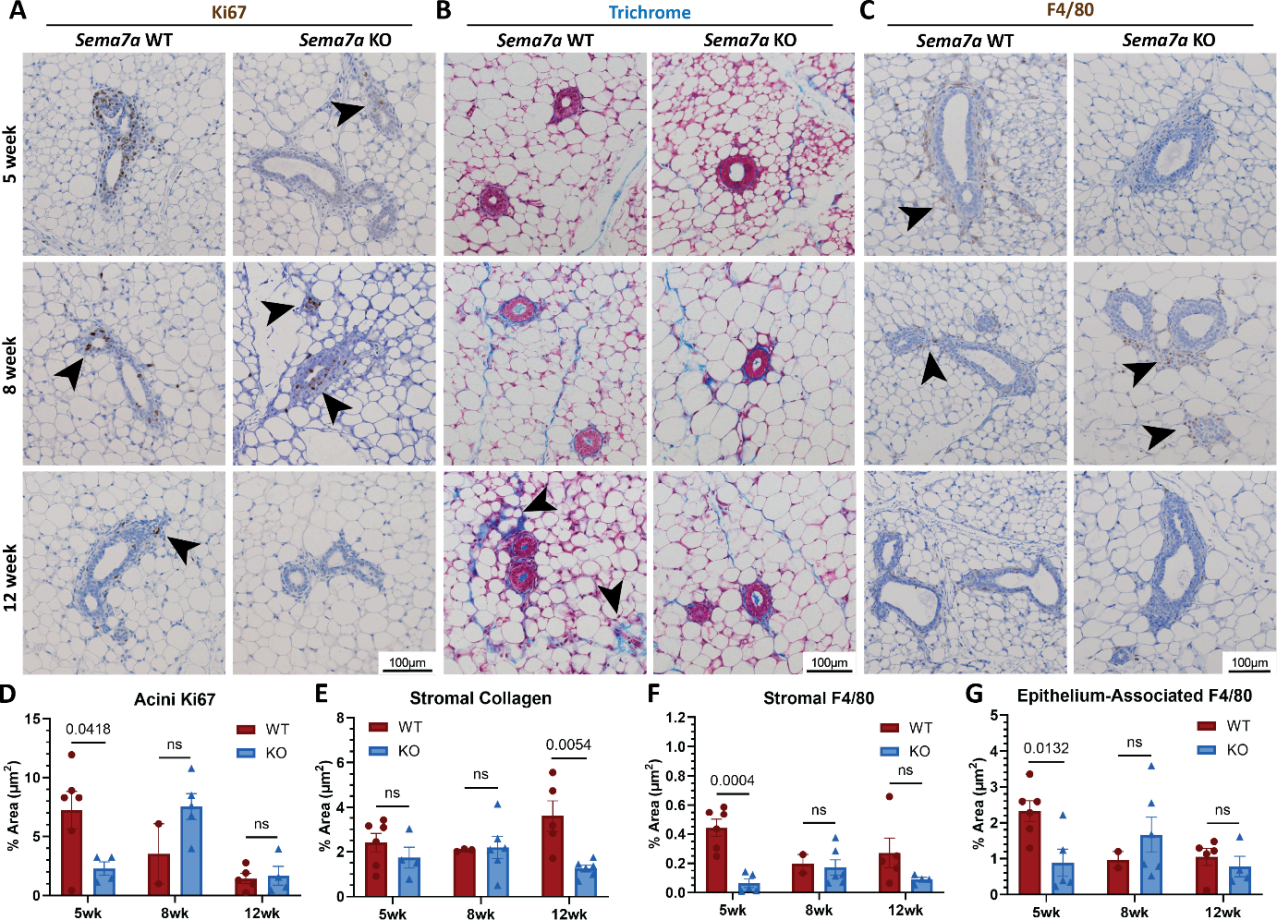
Knocking out SEMA7A decreases macrophages in the mammary gland during pubertal development. (A-C) Representative images from WT or *Sema7a* KO (*Sema7a*^*tm1Alk*^*/J*) mouse during pubertal development, at 5 weeks, 8 weeks, and 12 weeks of age; Ki67 (A), trichrome stain (B), and F4/80 (C). Black arrows denote observed changes in staining. (D) Quantification of Ki67 IHC in acini as a percent area of acini. Two-way ANOVA, p_timepoint_=0.0126, p_strain_=0.8256, p_interaction_=0.0095. (E) Quantification of stromal collagen as a percent area of the stroma. Two-way ANOVA, p_timepoint_=0.7432, p_strain_=0.0177, p_interaction_=0.0356. (F) Quantification of stromal F4/80 IHC as a percent area of the stroma. Two-way ANOVA, p_timepoint_=0.4382, p_strain_=0.0021, p_interaction_=0.0496. (G) Quantification of epithelium-associated F4/80 IHC as a percent area of epithelial structures. Two-way ANOVA, p_timepoint_=0.1192, p_strain_=0.3220, p_interaction_=0.0498. (D-G) p values determined by two-tailed t-test.

To characterize stromal changes in the *Sema7a* KO mouse during puberty, we analyzed cell proliferation, collagen volume and intensity, as well as macrophage localization by IHC. Since we found no changes in overall proliferation in the mammary gland during puberty between WT and *Sema7a* KO mice (Fig. 2H–I), we stratified proliferation by stroma, duct, and acini. We also observed no change in stromal proliferation by Ki67 expression between WT and KO mice throughout puberty (Fig. 3A, Supp Fig. 3A). However, we observed a decrease in proliferative ducts in KO compared to the WT at 5 weeks that was resolved at 8 and 12 weeks (Supp Fig. 3B). We also observed a decrease in proliferative acini in KO compared to WT at 5 weeks, an increase with proliferative acini in KO compared to WT at 8 weeks, and no change between WT and KO at 12 weeks (Fig. 3D).

Since collagen facilitates forward ductal elongation (47, 48, 63), we analyzed collagen via trichrome stain. We observed increased stromal collagen with the WT compared to KO at 12 weeks. (Fig. 3B, E) with no difference in epithelium-associated collagen or mean collagen intensity between WT and KO at any timepoint (Fig. 3B, Supp Fig. 3D-E). To investigate whether fibroblasts were contributing to the difference in stromal collagen, we analyzed αSMA in the stroma and observed a decrease in stromal αSMA at 8 weeks in the KO compared to WT which was also resolved by 12 weeks (Supp Fig. 3E).

We next identified localization of macrophages via F4/80 IHC and observed increased stromal macrophages in WT compared to KO at 5 and 8 weeks that was then decreased at 12 weeks (Fig. 3C, F). Furthermore, we observed increased epithelium-associated macrophages in the WT at 5 weeks, which was delayed until 8 weeks in the KO (Fig. 3C, G). For both stromal and epithelium-associated macrophages, the macrophage number in the KO did not reach the number of macrophages as the WT (Fig. 3F–G).

### SEMA7A blockade decreases DCIS invasion and blocks pro-invasive collagen phenotypes and protease activity

**Figure 4:**
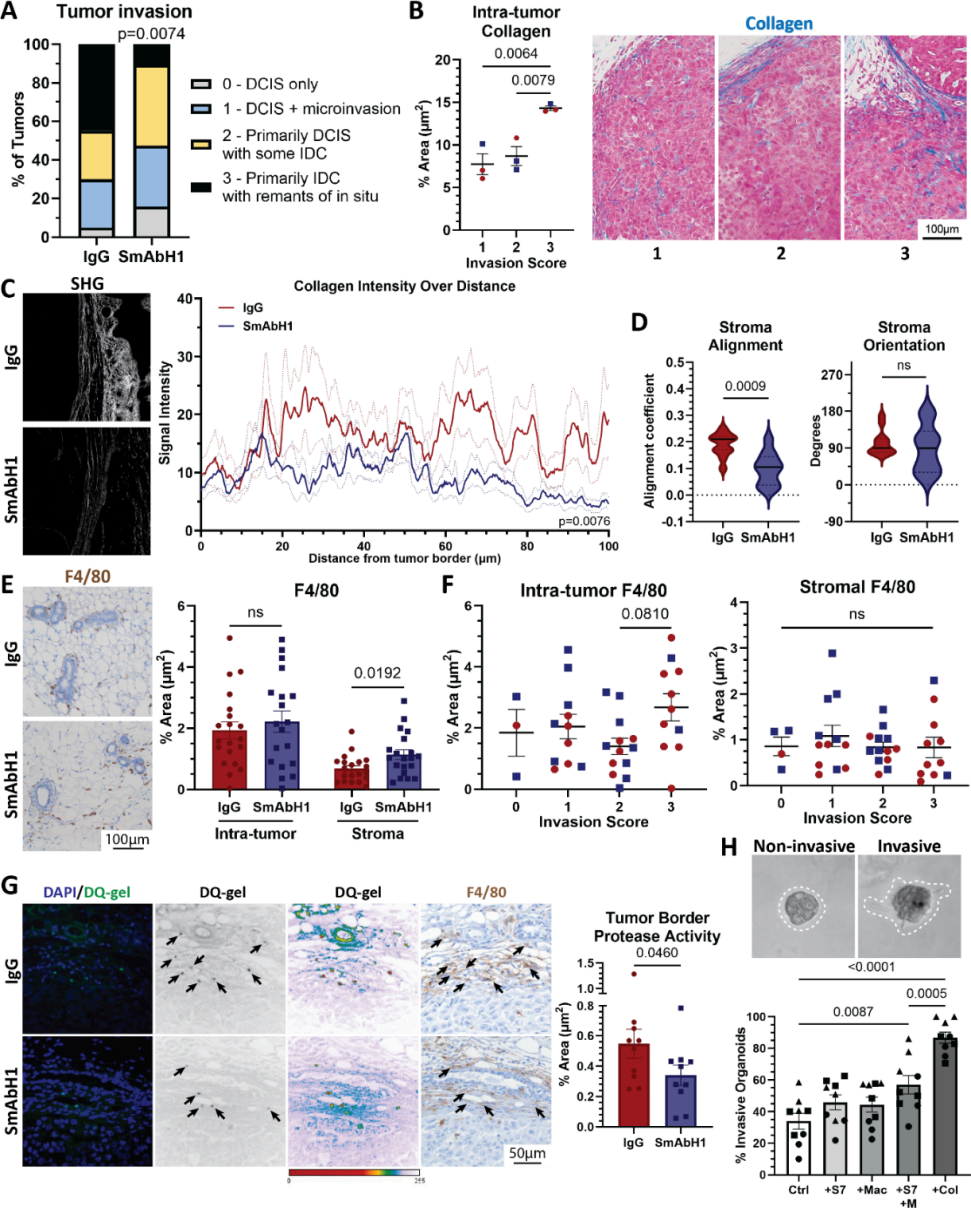
Treatment of DCIS tumors in mice with SmAbH1 decreases invasion of DCIS via stromal changes. (A) Quantification of tumor invasion scores per treatment group. IgG, n=20; SmAbH1, n=19. Significance by chi-square test. (B) Collagen expression in DCIS tumors. (left) Representative images of trichrome stain in DCIS tumors. (right) Quantification of collagen expression (blue stain) as a percent area in the tumor by invasion score. (C) Collagen intensity analysis by SHG. (left) Representative images of SHG of the tumor border and surrounding stroma. (right) Collagen intensity over distance from tumor border by treatment group. IgG, SmAbH1, n=3. (D) Quantification of stromal collagen alignment and orientation from SHG images of the stromal region outside the tumor border. IgG, n=12; SmAbH1, n=10. For orientation (right), F-test comparison of variance, p=0.0514. (F) F4/80 positive macrophage analysis by treatment group. (left) Representative images of F4/80 positive macrophages in the stroma outside the tumors. (right) Quantification of F4/80 positive macrophages as a percent area of tumor and stroma by treatment group. (F) Quantification of F4/80 positive macrophages as a percent area of the stroma (left) and intra-tumor (right) by invasion score. (G) In situ zymography (DQ-gelatin assay). (left) Representative images of tumor border with positive protease activity signals from left to right: fluorescent images, gray scale of DQ-gelatin fluorescent signal, histogram of high to low fluorescent signal, F4/80 IHC. Black arrows indicate colocalization of fluorescent signal with F4/80. (right) Quantification of protease activity at the tumor border by treatment. (H) DCIS organoid invasion assay co-cultured with RAW264.7 macrophages and/or 40ug/mL SEMA7A or 10% collagen. (top) Representative images of non-invasive and invasive organoids with organoid edge outlined. (bottom) Quantification of percent invasive MCF10DCIS.com organoids. (B, D, F) P values determined by two-tailed t-test. (E-G) P values determined by one-tailed t-test.

To test our hypothesis that a function blocking anti-SEMA7A antibody could decrease DCIS invasion via changes to macrophage localization and collagen structure, we treated mice with established MCF10DCIS tumors using our novel mouse monoclonal antibody that targets SEMA7A (SmAbH1) or an IgG control and measured tumor invasiveness at 4 weeks when MCF10DCIS tumors begin to progress to IDC (22). Using H&E sections of tumors with adjacent mammary gland tissue we utilized our previously established histologic invasion scoring system of DCIS and IDC (Supp Fig. 4A)(27). While tumor volume did not change with treatment (Supp Fig. 4B), we observed SmAbH1-treated tumors were less invasive than IgG-treated tumors. Specifically, only 10.5% of SmAb-treated tumors progressed to IDC (score 3), while 45% of IgG-treated tumors progressed to IDC (score 3)(Fig. 4A). 5% IgG-treated tumors scored as DCIS (score 0) compared to 15.5% of SmAbH1-treated tumors. We then analyzed collagen using a trichrome stain and observed no change in overall collagen in the tumor or the mammary fat pad (MFP) (Supp Fig. 4C). However, when tumors were stratified by invasion score, we observed increased collagen in the invasive tumors (score 3)(Fig. 4C). To quantify collagen surrounding the tumor, we analyzed collagen intensity and structure via second harmonic generation (SHG) in the tumor, tumor border, and stroma (Supp Fig. 4E). We found a decrease in collagen intensity in the tumor border with SmAbH1 treatment compared to IgG (Fig. 4C). We also quantified collagen alignment and orientation to measure parallel fibers and angled fibers, respectively. We observed a decrease in stromal collagen alignment with ShAbH1-treated tumors compared to IgG, suggesting SmAbH1-treated stromal collagen fibers are less parallel to each other (Fig. 4D). IgG-and SmAbH1-treated tumors had a 90-degree fiber orientation from a horizontal axis. SmAbH1-treated tumors had an increased range of stromal collagen orientation compared to IgG-treated tumors with an F-test variance p = 0.0514; therefore, a subset of SmAbH1-treated tumors had more angled fibers which did not appear in IgG-treated tumors (Fig. 2D). We did not observe a change in intratumoral macrophages between IgG and SmAbH1 (Fig. 4E). However, macrophages increased in the mammary gland stroma with SmAbH1-treated tumors compared to IgG (Fig. 4E). We then examined macrophage presence by invasion score and found macrophage presence in the stroma was also not significantly different with invasion (Fig. 4F). However, we did observe an increase in intratumoral macrophages with the most invasive tumors (score 3)(Fig. 2F). We also did not observe differences in macrophage localization at the tumor border with treatment (Supp Fig). Therefore, we hypothesized that macrophages at the tumor border may have decreased protease activity after treatment with SmAbH1. Thus, we measured protease activity at the tumor border using in situ zymography to show that SmAbH1 decreased protease activity at the tumor border (Fig. 4G), which also increased with invasion score (Fig. 4G). These areas with protease activity colocalized with high presence of F4/80 + macrophages, suggesting macrophages are expressing the proteases. Having previously shown that collagen increases DCIS organoid invasion in a 3D culture model (22), we then co-cultured RAW264.7 macrophages and MCF10DCIS organoids to test our hypothesis that macrophages promote DCIS invasion via SEMA7A-mediated protease activity. We observed that macrophages and SEMA7A separately did not significantly increase DCIS organoid invasion (Fig. 4H). However, macrophages and SEMA7A together increased DCIS invasion.

### SEMA7A promotes inflammatory cytokine expression in macrophages via a non-canonical beta-catenin signaling pathway

**Figure 5:**
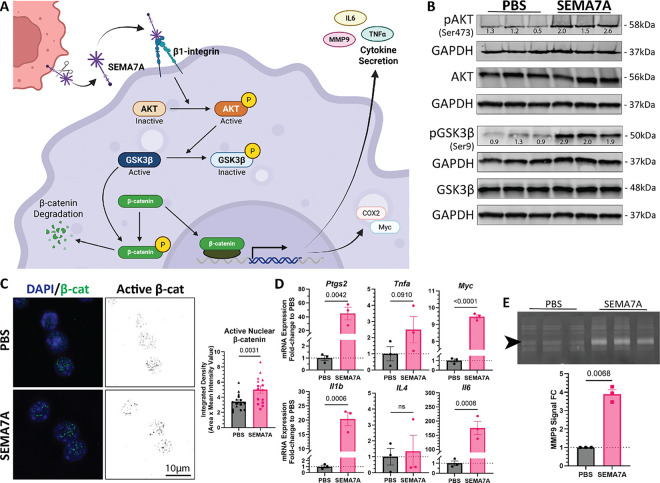
SEMA7A promotes protease and cytokine expression in RAW264.7 macrophages through a β1-integrin/AKT/GSK3β/β-catenin signaling pathway. (A) SEMA7A signaling cascade in macrophages. (B) Western blot of cell lysates from RAW264.7 macrophages treated with PBS or 40μg/mL SEMA7A. Phosphorylated protein signal values were normalized to GAPDH and total protein then fold change to average PBS. (C) Active β-catenin signal in RAW264.7 macrophages after treatment with PBS or SEMA7A. (left) Representative IF images of active β-catenin nuclear localization. (right) Quantification of nuclear active β-catenin signal. (D) qPCR analysis of β-catenin target genes in RAW264.7 macrophages as relative mRNA expression fold-change to PBS: *Ptgs2, Tnfa, Myc, Il1b, Il4*, and *Il6*. (E) (left) MMP zymography of conditioned media from RAW264.7 macrophages treated with PBS or SEMA7A, including MMP9 (black arrow, 82kDa). (right) Quantification of MMP9 signal intensity as fold change to average PBS. P values determined by one-tailed (C-D) or two-tailed (E) t-test.

To test our hypothesis that SEMA7A regulates macrophage function in the TME, we differentiated human THP-1 monocytes into macrophages with PMA and performed cytokine arrays on conditioned media from SEMA7A-treated THP-1 macrophages (Supp Fig. 5A-F). We identified an increase in signal from various cytokines that contribute to macrophage recruitment (GM-CSF, M-CSF)(Supp Fig. 5C) and macrophage-specific matrix remodeling (MCP-2/CCL2, MCP-3/CCL7)(Supp Fig. 5E). Additionally, many of these increased cytokines were pro-inflammatory, such as IL4 and IL6 (Supp Fig. 5D). We have previously established a relationship between SEMA7A signaling through proteinase kinase B (AKT) in cancer cells (64). Since AKT can regulate glycogen synthase kinase-3 beta (GSK3β) to non-canonically mediate inflammatory transcription factor β-catenin (65–67), we hypothesized that SEMA7A regulates non-canonical AKT/GSK3β/β-catenin signaling in macrophages (Fig. 5A). To test this, we treated mouse RAW264.7 cell line with exogenous SEMA7A or PBS control and evaluated phosphorylation of AKT and GSK3β, active β-catenin, and mRNA expression of downstream β-catenin targets. We observed expression of phosphorylated AKT (Ser473)(pAKT) and phosphorylated GSK3β (Ser9)(pGSK3β) in macrophages by immunoblot after treatment with SEMA7A (Fig. 5B) and we replicated this in THP-1 macrophages (Supp Fig. 6A). We also quantified active nuclear β-catenin (non-phosphorylated at Ser33/Ser37/Thr41) by immunofluorescence and found increased nuclear β-catenin after treatment with SEMA7A (Fig. 5C). Finally, we analyzed mRNA expression *of Ptgs2, Tnfa, Myc, Il1b, Il4*, and *Il6* to show that they were all increased in RAW264.7 and THP-1 mRNA after treatment with SEMA7A (Fig. 5D, Supp Fig. 6B). β-catenin is also upstream of matrix remodeling enzyme matrix metalloproteinase-9 (MMP9); thus, we measured MMP9 secretion in macrophages by MMP zymography to show that MMP9 is increased after treatment with SEMA7A (Fig. 5E, Supp Fig. 6C).

## Discussion

Approximately 50–80% of DCIS cases will not progress to IDC (10, 12, 13). This leads to overtreatment of patients diagnosed with DCIS and increased anxiety surrounding a DCIS diagnosis (4, 9, 68, 69). Currently, there is no definitively established biomarker for clinically predicting DCIS progression. However, the TME has been suggested as a major contributor to DCIS progression and therefore could offer predictive biomarkers (19, 21). We suggest that macrophages contribute to pro-invasive remodeling of collagen and may facilitate DCIS progression. Our results demonstrate that SEMA7A from epithelial cells and/or DCIS recruits macrophages and polarizes them towards having pro-inflammatory and matrix remodeling functions.

SEMA7A is positively correlated with CD68 + macrophages in DCIS and IDC patient tissues (Fig. 1) and loss of SEMA7A in our *Sema7a* KO mouse model has decreased macrophage infiltration during pubertal development (Fig. 3). These results are consistent with what we have previously shown in *Sema7a* KO mice where we observed decreased macrophage influx into the mammary gland during postpartum involution (34); together, these results support our overall hypothesis that SEMA7A regulation of macrophages is a normal mechanism of mammary morphogenesis that can be co-opted by tumor cells. Whether SEMA7A recruits peripheral macrophages or induces proliferation of resident macrophages remains unanswered by our current studies. However, we do reveal that loss of *Sema7a* delays pubertal development through slowed epithelial invasion that can be attributed to, in part, the decreased number of macrophages present. In support of this, the delay in elongation and altered TEB phenotype is similar to what is observed in a colony-stimulating factor-1 (CSF-1) knockout model of macrophages during pubertal development (38). Additionally, TEBs contain both macrophages and mammary stem cells (38, 70–72). Mammary stem cells are thought to give rise to epithelial cell populations during mammary morphogenesis, which could give rise to tumor cell populations (73). One molecule known to regulate mammary stem cell activity and organoid branching is TNFα (74), which we reveal is trending toward upregulated expression in SEMA7A-treated macrophages (Fig. 5D, Supp Fig. 6B) and has increased secretion in the cytokine array (Supp Fig. 5A, D). Therefore, SEMA7A-mediated TNFα expression by macrophages could also be promoting mammary stem cell outgrowth at the TEB. The cell line that we used for our model, MCF10DCIS, has also been shown to have a population of mammary stem cells (75); as such, blocking SEMA7A could decrease TNFα expression in macrophages and decrease mammary stem cell invasion in our DCIS model.

We also observe that macrophages are increased in patient samples of DCIS and IDC (Fig. 1) and are found increased in invasive tumors in mice (Fig. 4). Macrophages can contribute to breast tumor invasion by the remodeling of collagen in the mammary gland (35, 76–78). Studies have shown that bundled, organized collagen promotes breast cancer invasion (41, 54, 59, 79). Invasive tumor cells are thought to travel along organized collagen (80) and movement of epithelial cells along collagen stabilizes collagen fibrils (81). Furthermore, stabilized collagen promotes epithelial branching (81) and collagen orientation directs epithelial branching (48). Therefore, less organized collagen around SmAbH1-treated tumors makes cell invasion less attainable. This is likely due to SEMA7A regulating macrophage-mediated collagen remodeling such as promoting protease activity at the tumor border which was decreased with SmAbH1 treatment. Furthermore, previous studies from our lab have suggested that collagen fibers in this DCIS model, and in organoid models, can serve to upregulate COX-2, which can then facilitate mechanisms of cellular invasion (22). COX-2 can also drive expression of SEMA7A, which then feeds back to stimulate collagen production by fibroblasts (22, 24, 27). Our results now support a role for macrophages in this feedback mechanism by showing that the collagen in the TME is altered in a SEMA7A-dependent manner and that the macrophages respond by increasing *Ptgs2* mRNA—the gene that encodes for COX-2 protein. This is further supported by previous studies that show that COX-2 inhibition with NSAIDs can alter monocyte/macrophage differentiation (82) and block tumor cell invasion.

COX-2 is also a marker of alternatively-activated, or M2-like, macrophages (83, 84) along with MMP9 (85). Moreover, collagen stabilization through crosslinking polarized macrophages toward an M2-like phenotype (86). While M2-like macrophages are typically known for having anti-inflammatory signaling, our results demonstrate that SEMA7A promotes a pro-inflammatory phenotype in mammary macrophages. The pro-inflammatory cytokines that are increased after treatment with SEMA7A can lead to matrix remodeling in the TME, which can further support the invasion of the tumor cells (87–90) to promote breast cancer progression (91–97). Furthermore, some of these cytokines can activate β-catenin, suggesting a potential positive feedback loop after SEMA7A stimulation (98–101). Therefore, SEMA7A could be polarizing macrophages toward a regulatory macrophage phenotype, which is a subset of M2-like macrophages known as M2b. Regulatory M2b macrophages express pro-inflammatory cytokines such as IL6 and TNFα and have been found to be a subset of tumor-associated macrophages (TAMs) that promote growth, invasion, and recurrence of tumors (102). More studies focused on M2b macrophages in the mammary gland and polarization of macrophages by SEMA7A would provide insight into the role of SEMA7A-programmed pro-inflammatory macrophages in breast cancer.

Semaphorins have been implicated in canonical Wnt/β-catenin signaling in epithelial cells and fibroblasts through plexin receptor signaling (103). SEMA7A has been shown to activate β-catenin signaling in vascular smooth muscle cells and promotes expression of targets c-myc, cyclin D1, and MMP7, although this was found to be through intracellular SEMA7A signaling and not through receptor binding (104). Our studies are the first to link SEMA7A receptor signaling and β-catenin signaling to epithelial cell invasion in the mammary gland. Wnt/β-catenin signaling, which plays a major role in embryonic mammary epithelium development (105, 106), is also crucial for organoid branching of mammary epithelium (107–109). This mammary epithelial expansion is largely regulated by Wnt-mediated actin/cytoskeleton reorganization (107, 108). Additionally, cytoskeletal dynamics are regulated during TEB elongation and epithelial cell invasion (110, 111) and Wnt/β-catenin signaling from fibroblasts has been shown to regulate epithelial branching (107), suggesting that paracrine signaling from stromal cells can promote epithelial invasion. Furthermore, Wnt/β-catenin signaling promotes activation of mammary stem cells in both normal development and tumor models (105, 112–115), including DCIS (116). In DCIS, β-catenin expression is increased and conserved with IDC where Wnt/β-catenin is highly activated (117) and GSK3b protein expression is decreased in IDC tissues, suggesting accumulation of β-catenin (118). Finally, activation of Wnt/β-catenin is thought to activate an epithelial-to-mesenchymal transition (EMT)(117, 119, 120), which is a suggested mechanism for DCIS transition to IDC (121–123). While further studies are required to establish a role for Wnt/β-catenin signaling in regulating intrinsic DCIS cell invasion, SEMA7A-mediated β-catenin signaling in macrophages could be regulating migration through increased cytoskeletal dynamics, as well as matrix remodeling and cell invasion through EMT activation.

## Conclusion

We demonstrate that macrophage-mediated matrix remodeling is facilitated in part by a collagen, COX-2, SEMA7A signaling axis initiated from epithelial and tumor cells in the mammary gland. These SEMA7A-programmed matrix remodeling macrophages have increased protease activity and cytokine secretion which can restructure collagen toward a pro-invasive phenotype. Our findings help further understand SEMA7A in the mammary gland, both during normal development and with DCIS progression, specifically focusing on changes to the TME, and are the first to recognize a regulating role for SEMA7A during pubertal development. We reveal that SEMA7A regulates normal pubertal development in part by increasing the type of macrophages that are likely promote epithelial and tumor cell invasion. During DCIS progression, SEMA7A may stimulate macrophage-mediated collagen remodeling around tumors. Our results support further investigation into increased mammary gland expression of SEMA7A with organized peri-tumoral collagen as potential biomarkers for DCIS cases at high risk for invasion. Targeting SEMA7A, such as with our inhibitor SmAbH1, may also be a mechanism for slowing or reversing pro-invasive mammary gland changes in patients that are at high risk. We did not observe any evidence of toxicity with SmAbH1. Furthermore, blocking SEMA7A has potential for reversing pro-inflammatory signaling in tumors. This, along our previous analysis of the contribution of COX-2 to this signaling mechanism and evidence that NSAIDs can also alter collagen in the mammary gland and the TME, suggest that co-targeting of SEMA7A and COX-2 could also be explored (22). By identifying patients at high risk for invasion, DCIS patients with low risk could forego more invasive treatments and have decreased psychological distress upon diagnoses. Through identifying and targeting potential biomarkers that regulate the TME, such as SEMA7A, collagen, and COX-2, we can further our understanding of contributing factors to DCIS invasion and begin to address overtreatment and anxiety in DCIS patients with safer, less toxic, interventions.

## Data Availability

Dataset from TCGA from the Early Stage Breast Tumor cohort is available at the University of Alabama at Birmingham MammOnc-DB at http://resource.path.uab.edu/MammOnc-Home.html. All author-generated datasets used and analyzed are available from the corresponding author on reasonable request.

## Abbreviations

AKT: Protein kinase B
COX-2: Cyclooxygenase-2
CSF-1: Colony-stimulating factor-1
DCIS: Ductal carcinoma in situ
EMT: Epithelial-to-mesenchymal transition
GSK3β: Glycogen synthase kinase-3 beta
IDC: Invasive ductal carcinoma
IL: Interleukin
KO: Knock out
MFP: Mammary fat pad
MMP: Matrix metalloproteinase
SEMA7A: Semaphorin 7a
TAM: Tumor associated macrophage
TEB: Terminal end bud
TMA: Tumor microarray
TME: Tumor microenvironment
TNFα: Tumor necrosis factor alpha
US: United States
WT: Wild type
